# Association between pet ownership and mental health in university students with borderline personality disorder symptoms

**DOI:** 10.1186/s12888-026-07805-8

**Published:** 2026-01-16

**Authors:** Kanyarat Khattiya, Tinakon Wongpakaran, Nahathai Wongpakaran

**Affiliations:** 1 Faculty of Medicine, Chiang Mai University, Chiang Mai, Thailand; 2 Department of Psychiatry, Faculty of Medicine, Chiang Mai University, Chiang Mai, Thailand

**Keywords:** Borderline personality disorder, Pets, Mental health, University students

## Abstract

**Background:**

Approximately 9.7% of university students exhibit symptoms of borderline personality disorder (BPD), characterized by disturbed self-identity, low self-esteem, and emotion dysregulation. Caring for a pet may enhance self-esteem and provide a sense of purpose, potentially benefiting those with BPD.

**Methods:**

This study aimed to explore the relationship between pet ownership and mental health in 346 university students with BPD symptoms. Participants were classified as pet owners or non-pet owners. Mental health outcomes were assessed using validated self-report instruments, including the Outcome Inventory-21 (OI-21), the Rosenberg Self-Esteem Scale–Thai Revised (RSES-TR), the Revised Thai version of the Multidimensional Scale of Perceived Social Support (R-Thai MSPSS), the Thai version of the 10-item Perceived Stress Scale (T-PSS-10), and the Inner Strength-Based Inventory (iSBI).

**Results:**

No significant differences were observed between the two groups in adverse mental health outcomes. However, pet owners demonstrated higher levels of inner strengths, including truthfulness (t (240) = 4.10, *p* < 0.001), wisdom (t (240) = 3.72, *p* < 0.001), generosity (t (242) = 2.16, *p* = 0.032), tolerance (t (242) = 2.44, *p* = 0.016), determination (t (237) = 2.43, *p* = 0.017), and loving-kindness (t (242) = 2.04, *p* = 0.043). Notably, dog owners reported lower levels of anxiety (t (237) = -2.40, *p* = 0.017), depression (t (243) = -2.03, *p* = 0.043), and somatization (t (244) = -2.56, *p* = 0.025), while also scoring higher in self-esteem (t (242) = 2.15, *p* = 0.032) compared to non-pet owners. In contrast, no significant differences in mental health outcomes were found between cat owners and non-pet owners.

**Conclusion:**

These findings suggest that pet ownership, especially ownership of dogs, may be positively associated with mental health among university students with BPD symptoms. Future research should explore intervention-based approaches to validate these results.

## Background

Borderline Personality Disorder (BPD) is a complex psychiatric condition characterized by emotional instability, impulsivity, an unstable self-image, and chronic feelings of emptiness, all of which contribute to low self-esteem [1]. Recent longitudinal data from a five-year study (2017–2021) of over 12,700 U.S. college students found that the prevalence of significant borderline personality disorder (BPD) symptoms, defined by a score of ≥ 5 on the McLean.

Screening Instrument for Borderline Personality Disorder, increased annually by 24%, with students enrolled during the COVID-19 pandemic having 36% higher odds of meeting the symptom threshold compared to pre-pandemic students [2]. In Thai university students, the prevalence has been identified at 6.4%, as measured by the Screening instrument for borderline personality disorder (SI-Bord) with a cut-off score of 9 or higher [3].

BPD is closely associated with other mental health conditions. A large-scale study conducted in 2014 involving 34,481 U.S. adults reported that individuals with BPD were found to have significantly higher lifetime rates of mood disorders (83%), anxiety disorders (85%), substance use disorders (78%), post-traumatic stress disorder (PTSD) (30%), and other personality disorders (53%) [4]. The university student age group, typically between 18 and 29 years, is a period of “emerging adulthood,” marked by substantial instability in relationships, work, and living situations, all of which heighten the risk of mental health disorders [5]. Although these transitions are developmentally typical, individuals without secure attachment may be more vulnerable to negative mental health outcomes when facing such stressors. Suicide behavior is especially prevalent among individuals with BPD, with over 75% attempting suicide, and about 10% ultimately dying by suicide, as reported in an earlier study [6]. Notably, BPD has been shown to have a greater impact on suicide ideation than depressive symptoms alone, particularly in those who exhibit self-injurious behaviors and chronic feelings of emptiness [7–8].

Attachment theory offers a valuable framework for understanding the interpersonal difficulties faced by individuals with BPD [9]. It proposes that early experiences with caregivers form internal working models that guide expectations and behavior in relationships throughout life. Insecure attachments—often stemming from inconsistent or traumatic caregiving—are believed to contribute to core features of BPD, such as emotion dysregulation, interpersonal instability, and chronic fears of abandonment [9].

Both insecure attachment and BPD contribute to dysfunctional emotional and relational processes, though they manifest at different stages of social interactions. Insecure attachment is linked to heightened negative perceptions of others and difficulties with emotion regulation, whereas BPD is more strongly associated with interpersonal dysfunction during interpersonal interactions. As a result, individuals with BPD often have smaller and less diverse social networks, along with lower social functioning, particularly in areas such as interpersonal communication and prosocial behavior, for example, research has shown that individuals with BPD display significantly reduced prosocial behaviors, such as helping or cooperating with others, compared to both healthy controls and clinical comparison groups [10–11]. These impairments, along with an unstable sense of self, contribute to heightened feelings of loneliness, which are reported at significantly higher levels in people with BPD compared to healthy individuals. Consequently, individuals with BPD frequently exhibit heightened sensitivity to abandonment and an increased need for reassurance in close relationships, constantly seeking support from others to avoid feelings of loneliness [12–14].

Secure attachments are often nurtured through positive family relationships, safe environments, and supportive connections with peers and adult role models. These attachments provide a sense of security and trust, forming the basis for sustained emotion regulation and interpersonal relationships [52]. Interestingly, individuals who seek secure attachments may also form such bonds with pets, suggesting that attachment needs can extend beyond human relationships.

Research has shown that pets can serve as a valuable source of social support, particularly for individuals with attachment difficulties. For example, animals living in foster homes have been found to play a crucial role in helping children form secure attachments [15]. In older adults, pet ownership has been associated with reduced loneliness, increased social interaction, and a greater sense of purpose, all of which contribute to improved resilience against mental health disorders; for instance, a study by Hui Gan and colleagues found that older adults with pets were 36% less likely to experience depressive symptoms and reported significantly lower loneliness scores than non-pet owners [16]. A study conducted in the UK found that young adults perceive pet ownership, particularly dogs and cats, as beneficial for managing symptoms of anxiety and depression [17]. Furthermore, approximately 40% of pet owners report receiving social support through connections made through their pets, such as activities like dog-walking in local parks, visiting pet-friendly cafés, or participating in community pet events, all of which facilitate casual interactions and long-term friendships [18]. Higher levels of perceived social support have been shown to reduce suicide ideation and enhance self-esteem, particularly among dog owners [19].

In addition to alleviating psychological stress, research suggests that pets also provide physiological benefits. Pet therapy has been associated with significant improvements in physiological markers, including reduced heart rate [20]. For individuals with BPD, pets may serve an even more substantial role. Studies indicate that pets provide opportunities for social engagement, participation in meaningful activities, and the development of coping strategies [21]. Additionally, they may support the formation of secure attachments, which is particularly important for individuals who struggle with interpersonal relationships [21], possibly by offering a consistent and non-judgmental emotional bond that mirrors the concept of a “secure base” in attachment theory [14, 17, 20].

Despite the potential benefits of pet ownership, research on its impact on individuals with BPD remains limited, particularly among emerging adults in university settings. This study aimed to explore the relationship between pet ownership and mental health in university students with BPD symptoms. Specifically, it compared both positive mental health outcomes—such as inner strength, social support, and self-esteem—and negative mental health outcomes—such as anxiety, depression, somatization, interpersonal difficulties, and perceived stress—between students with and without pets.

## Methods

This study used a cross-sectional design to examine the relationship between pet ownership and mental health outcomes among university students exhibiting symptoms of BPD. Participants were recruited from universities across Thailand and screened using the SI-Bord, a tool designed to identify BPD symptoms.

### Participants

The study included university students in Thailand aged 20–30 years who were enrolled in undergraduate, master’s, doctoral, or diploma programs. The lower age limit of 20 was selected to ensure that all participants were legally recognized as adults under Thai law, allowing them to provide informed consent without requiring parental permission. Although university students may begin their studies as early as age 18, individuals under 20 are considered minors in Thailand. The upper age limit of 30 was used to align with the definition of emerging adulthood.

University students were selected as the target population because they are particularly vulnerable to mental health challenges due to the transition from adolescence to adulthood, academic stress, and adjustment to independent living. Studies have shown that the prevalence of anxiety, depression, and emotional distress is disproportionately high in college populations, with many institutions reporting a surge in the use of campus mental health services. In response, emotional support animals have become increasingly popular in university settings, particularly in the U.S., where institutions are adapting policies to accommodate them in student housing as a mental health support strategy [53–54]. Therefore, examining pet ownership and its associations with psychological well-being in a university sample offers a timely and relevant contribution to the growing body of research on non-traditional mental health interventions in emerging adults.

Of the 1,244 individuals initially screened, 346 met all inclusion criteria and were included in the final analysis, comprising 173 in the pet-owner group and 173 in the non-pet-owner group. Participants were required to be fluent in Thai to ensure comprehension of the study materials. For the pet ownership group, participants needed to own at least one pet and score greater than seven on the SI-Bord scale, which assesses BPD symptoms. The inclusion criteria for the non-pet group were identical, except that those participants did not own pets. To ensure adequate statistical power and facilitate group comparisons, we intentionally recruited an equal number of participants in the pet- and non-pet-owner groups (*n* = 173 per group). This matched-group design was based on an a priori sample size calculation to compare two independent means, with 80% power and a significance level of 0.05.

Exclusion criteria for both groups included a diagnosis of psychiatric disorders involving psychotic symptoms, bipolar disorder, or substance use disorder. Participants were also excluded if they had a history of substance use within 24 h before the study. To assess this, participants were informed of the exclusion criteria during the consent and pre-screening stage. They were asked to confirm their eligibility, including the absence of recent substance use. Additionally, individuals who cared for others’ pets but were not the primary pet owners were deemed ineligible to participate.

Participants in the pet owner group were also asked to report their interaction style with their pets, categorized as either Human–Animal or Human–Human. The Human–Animal style reflects typical pet-owner relationships, focusing on care and affection, for example an owner might say: “Sit, boy!” or “Good dog!”, while the Human–Human style involves treating pets as human companions or family members—for example, talking to them, using personal names, or treating them like a child. The same owner might say: “Max, sweetie, are you feeling okay today?” or “Come cuddle with mommy”.

**Fig. 1:**
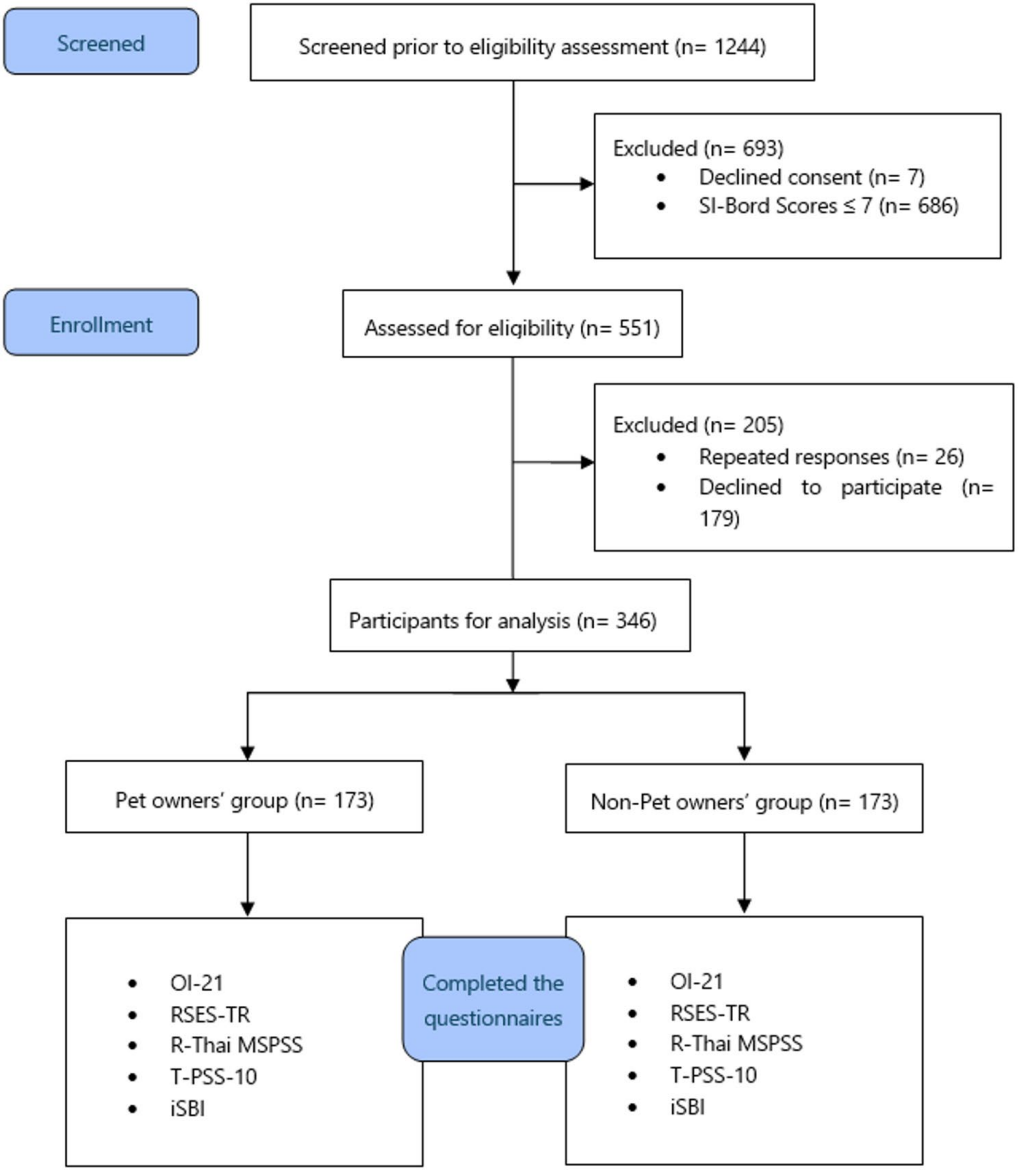
Overall study design flowchart. Legend: OI-21 = Outcome Inventory-21, RSES-TR = Rosenberg Self-Esteem Scale-Thai Revised, R-Thai MSPSS = Revised Thai version of the Multidimensional Scale of Perceived Social Support, T-PSS-10 = Thai version of the 10-item Perceived Stress Scale, iSBI = Inner Strength-Based Inventory

Participants were recruited via convenience sampling through online platforms (e.g., university websites, social media groups) and physical advertisements posted on university campuses and at psychiatric outpatient clinics across Thailand. While the exact number of participating universities was not recorded, participants were recruited from multiple institutions across Thailand to ensure geographic and academic diversity. Interested individuals were first screened online using the SI-Bord tool, and eligible participants were invited to complete a comprehensive survey (Fig. 1). Participants could complete the survey online or on paper, and all responses were anonymized. Informed consent was obtained from all participants, and ethical approval was obtained from the Faculty of Medicine, Chiang Mai University.

### Instruments

This study used several validated instruments, including the SI-Bord, Outcome Inventory-21 (OI-21), Rosenberg Self-Esteem Scale-Thai Revised (RSES-TR), Revised Thai version of the Multidimensional Scale of Perceived Social Support (R-Thai MSPSS), the Thai version of the 10-item Perceived Stress Scale (T-PSS-10), and the Inner Strength-Based Inventory (iSBI).

1. **SI-Bord**: The SI-Bord is a 5-item scale based on DSM-5 criteria for BPD, scored on a 4-point Likert scale (0 = never, 3 = often). It measures BPD symptoms such as abandonment avoidance, interpersonal difficulties, identity disturbance, suicide behavior, and emotional instability. The scores range from 0 to 15. The higher score indicates a higher level of BPD symptoms. A cutoff score > 7 is used to identify significant BPD symptoms. The SI-Bord has a sensitivity of 75.00% and a specificity of 73.08%.
2. **Outcome Inventory-21 (OI-21)**: The OI-21 measures anxiety, depression, somatization, and interpersonal difficulties across 21 items. This 21-item inventory utilizes a Likert scale ranging from 0 (never) to 4 (almost always), with total scores ranging from 0 to 48. Higher scores indicate greater symptom severity. Its depression subscale has a sensitivity of 82.72% and a specificity of 78.35%. Cronbach’s alpha for the entire scale is 0.92, and subscale alphas range from 0.80 to 0.87 [22]. In this study, Cronbach’s alpha for the scale was 0.93.
3. **Rosenberg Self-Esteem Scale-Thai Revised (RSES-TR)**: The RSES-TR is a tool to assess self-esteem. The questionnaire consists of 10 items rated on a 4-point scale, ranging from 1 (Strongly Disagree) to 4 (Strongly Agree). Total scores range from 10 to 40, with higher scores indicating higher self-esteem. Cronbach’s alpha is 0.86 [23]. In this study, Cronbach’s alpha for the scale was 0.86.
4. **Revised Thai version of the Multidimensional Scale of Perceived Social Support (R-Thai MSPSS)**: The R-Thai MSPSS evaluates perceived social support through 12 items, scored from 1 (strongly disagree) to 7 (strongly agree). Total points range between 12 and 84 points. Higher scores reflect more substantial social support, and Cronbach’s alpha is 0.91 [24]. In this study, Cronbach’s alpha for the scale was 0.94.
5. **Thai version of the 10-item Perceived Stress Scale (T-PSS-10)**: The T-PSS-10 measures perceived stress through 10 items scored from 0 (never) to 4 (very often). The total score ranges from 0 to 40. Higher scores indicate greater perceived stress, with a Cronbach’s alpha of 0.85 [25]. In this study, Cronbach’s alpha for the scale was 0.78.
6. **Inner Strength-Based Inventory (iSBI)**: The iSBI assesses inner strength through 10 items measuring attributes such as truthfulness, perseverance, wisdom, generosity, precept, meditation, tolerance, equanimity, determination, and loving-kindness. Each item is rated on a 5-point scale, with scores ranging from 10 to 50. Higher scores indicate greater inner strength. Cronbach’s alpha for the iSBI is 0.86 [26]. This study did not calculate Cronbach’s alpha as each was treated as an independent variable.

### Statistical analysis

Descriptive statistics, including means, standard deviations, medians, and frequencies, were used to summarize demographic characteristics and psychological outcomes.

Independent t-tests were used to compare mean scores between pet owners and non-pet owners, with effect sizes reported. Normality assumptions for continuous outcomes were assessed using the Shapiro-Wilk test, Q-Q plots, and histograms. In cases where the Shapiro-Wilk test was statistically significant, indicating a deviation from normality, but Q-Q plots and histograms suggested approximate normal distribution, we considered the visual inspection alongside the test statistic. Given the relatively large sample size and absence of pronounced skewness or outliers, parametric analyses were used. Key findings were also confirmed with non-parametric tests to ensure robustness. The chi-square test was employed for categorical variables. Pearson or Spearman correlation coefficients, as appropriate based on distributional properties, were calculated to examine associations between pet ownership and mental health outcomes. Statistical significance was set at *p* < 0.05. However, for the t-test, given that multiple outcomes were analyzed, we adjusted the significance threshold using the Bonferroni correction. P-values were considered statistically significant only if they remained below the adjusted threshold after correction for multiple comparisons. With five 21 outcomes, statistical significance was set at *p* < 0.0024 for each test (0.05/21) after Bonferroni correction.

All analyses were conducted using IBM SPSS version 26 (SPSS Inc.).

## Results

Table 1 shows that the study included a total of 346 participants, and most were female (74.6%). Among pet owners, most were female as well (39.0%). However, there was no statistically significant difference in gender distribution between pet owners and non-pet owners (X² (1) = 2.20, *p* = 0.139). The mean age of all participants was 21.6 years (SD = 2.24), and there was no significant difference between the groups (t (344) = 1.13, *p* = 0.259). Among pet owners, most of the participants were 2nd-year students (47%). Most participants were single (98.0%). The number of pets further categorized pet owners; most (45.1%) had one pet.

**Table 1:** Sociodemographic data: comparison between pet and non-pet groups

Variables	All (*n* = 346)	Pet (*n* = 173)	No Pet (*n* = 173)	Test Difference
Sex				X² (1) = 2.20, *p* = 0.139
Male	88 (25.4%)	38 (11.0%)	50 (14.5%)	
Female	258 (74.6%)	135 (39.0%)	123 (35.5%)	
Age	21.6 ± 2.24	21.74 ± 2.34	21.47 ± 2.13	*t* (344) = 1.13, *p* = 0.259, (95%CI -0.20, 0.74)
Year of Study				X² (4) = 2.79, *p* = 0.594
1 year	33 (9.5%)	16 (4.6%)	17 (4.9%)	
2 years	85 (24.6%)	47 (13.6%)	38 (11.0%)	
3 years	95 (27.5%)	43 (12.4%)	52 (15.0%)	
4 years	75 (21.7%)	35(10.1%)	40 (11.6%)	
More than 4 years	58 (16.8%)	32 (9.2%)	26 (7.5%)	
Marital status				X² (2) = 3.74, *p* = 0.154
Single	339 (98.0%)	172 (49.7%)	167 (48.3%)	
Married	6 (1.7%)	1 (0.3%)	5 (1.4%)	
Divorced				
Widowed	1 (0.3%)	0 (0.0%)	1 (0.3%)	
The number of pets				*-*
1	78 (22.6%)	78 (45.1%)	N/A	
2	38 (11%)	38 (22%)		
More than 2	57 (16.4%)	57 (32.9%)		

Participants reported a variety of pet types. The most common were cats (64.2%) and dogs (43.9%), followed by fish (8.1%), rodents (5.2%), rabbits (2.9%), birds (2.3%), and reptiles, including turtles (2.3%). Less common pets included cows, praying mantises, spiders, and chickens (each 0.6%). Pet owners also reported their preferred human–pet interaction style; the majority identified with a “Human–Human” style (82.7%), and the remainder with a “Human–Animal” style (17.3%) (Table 2).

**Table 2:** Characteristics of the pet group

	*n*	%
The number of pets		
1	78	45.1
2	38	22.0
More than 2	57	32.9
The type of pet		
Dog	76	43.9
Cat	111	64.2
Bird	4	2.3
Fish	14	8.1
Rabbit	5	2.9
Rodent	9	5.2
Reptile (including Turtle)	4	2.3
Other		
– Cow	1	0.6
– Praying mantis	1	0.6
– Spiders	1	0.6
– Chickens	1	0.6
Human-pet interaction style		
Human - Animal	30	17.3
Human - Human	143	82.7

Assumptions of normality were assessed and deemed adequately met based on both visual and statistical analyses; see Methods for details. We then conducted independent t-tests to compare anxiety and depression scores between pet owners and non-pet owners.

Negative mental health outcomes are presented in Table 3, comparing pet and non-pet groups. The average anxiety (t (336) = -1.35, *p* = 0.177), depression (t (341) = -1.00, *p* = 0.319), somatization (t (338) = -0.94, *p* = 0.349), and overall OI-21 score (t (326) = -0.92, *p* = 0.357), were slightly lower for pet owners; however, these differences were not significant. Furthermore, the two groups had identical average Perceived Stress Scale (PSS) scores (t (335) = 0.02, *p* = 0.988).

**Table 3:** Negative mental health variables comparing between the pet and no pet groups

Outcome	Pet (*n* = 173)	No pet (*n* = 173)	Test Difference				
			t	df	*p*-value	95% Confidence Interval	
						Lower	Upper
OI-21							
Anxiety	13.84 ± 4.77	14.54 ± 4.86	-1.35	336.00	0.177	-1.73	0.32
Depression	8.42 ± 4.35	8.86 ± 3.87	-1.00	341.00	0.319	-1.32	0.43
Interpersonal difficulty	8.74 ± 3.34	8.43 ± 3.36	0.86	343.00	0.390	-0.40	1.02
Somatization	9.8 ± 5.15	10.3 ± 4.56	-0.94	338.00	0.349	-1.53	0.54
Total score	40.76 ± 15.13	42.24 ± 13.92	-0.92	326.00	0.357	-4.64	1.68
PSS	22.54 ± 6.61	22.54 ± 5.24	0.02	335.00	0.988	-1.27	1.29

OI-21 = Outcome Inventory-21, PSS = Perceived Stress Scale, *df* = degree of freedom

Regarding positive mental health outcomes (Table 4), pet owners reported higher self-esteem (t (342) = 0.96, *p* = 0.338) and total perceived social support scores (t (336) = 0.54, *p* = 0.591), although these differences were not statistically significant. However, for iSBI, pet owners scored significantly higher on the “Wisdom” (t (343) = 2.13, *p* = 0.034) and “Precept” (t (342) = 2.55, *p* = 0.011) than non-pet owners.

**Table 4:** Positive mental health variables compared between the pet and no pet groups

Outcome	Pet (*n* = 173)	No pet (*n* = 173)	Test Difference					
			t	df	*p*-value	95% Confidence Interval		Cohen’s d
						Lower	Upper	
RSES	26.14 ± 5.25	25.61 ± 5.01	0.96	342.00	0.338	-0.56	1.62	0.104
MSPSS								
Significant Other	18.60 ± 7.00	17.88 ± 6.88	0.97	341.00	0.334	-0.75	2.20	0.105
Friends	18.25 ± 6.27	18.40 ± 6.33	-0.22	340.00	0.824	-1.49	1.19	-0.024
Family	17.88 ± 6.62	17.43 ± 6.72	0.62	343.00	0.537	-0.97	1.86	0.067
Total	54.67 ± 16.70	53.68 ± 17.35	0.54	336.00	0.591	-2.65	4.64	0.059
iSBI variables								
Truthful	3.21 ± 1.26	3.18 ± 1.28	0.25	343.00	0.806	-0.24	0.30	0.027
Perseverance	2.49 ± 1.06	2.33 ± 0.10	1.42	342.00	0.156	-0.06	0.38	0.154
Wisdom	3.09 ± 1.20	2.81 ± 1.18	2.13	343.00	0.034	0.02	0.52	0.230
Generosity	3.59 ± 1.21	3.37 ± 1.32	1.60	340.27	0.111	-0.05	0.49	0.173
Precept	2.99 ± 1.27	2.65 ± 1.16	2.55	342.00	0.011	0.08	0.59	0.276
Meditation	1.53 ± 0.87	1.44 ± 0.70	1.03	342.00	0.303	-0.08	0.25	0.111
Tolerance	3.23 ± 1.15	3.10 ± 1.11	1.04	343.00	0.299	-0.11	0.37	0.112
Equanimity	3.01 ± 1.06	2.88 ± 0.97	1.17	343.00	0.243	-0.09	0.34	0.126
Determination	3.12 ± 1.10	2.91 ± 1.13	1.74	341.00	0.082	-0.03	0.45	0.188
Loving-Kindness	3.26 ± 1.23	3.20 ± 1.24	0.47	343.00	0.638	-0.20	0.32	0.051

RSES = Rosenberg Self-Esteem Scale, MSPSS = Multidimensional Scale of Perceived Social Support, iSBI = Inner Strength-Based Inventory, df = degree of freedom

Table 5 presents a comparison of psychological variables between dog owners and non-pet owners. Dog owners reported significantly lower levels of anxiety (t (237) = -2.40, *p* = 0.017), depression (t (243) = -2.03, *p* = 0.043), and somatization (t (239) = -2.26, *p* = 0.025) than non-pet owners. Regarding RSES, dog owners reported significantly higher self-esteem than non-pet owners (t (242) = 2.15, *p* = 0.032). No significant differences were observed between dog owners and non-pet owners in perceived social support from any sources. Dog ownership was also associated with higher inner strength (t(242) = 1.99, *p* = 0.047) and significantly higher scores on perseverance, wisdom, generosity, determination, and loving-kindness.

**Table 5:** Positive and negative mental health variables comparing the dog and no pet groups

Outcome	Dog Only Owners (*n* = 76)	No Pet Owners (*n* = 170)	Test Difference					
			t	df	*p*-value	95% Confidence Interval		Cohen’s d
						Lower	Upper	
OI-21								
Anxiety	13.05 ± 4.60	14.64 ± 4.73	-2.404	237	0.017*	-2.88	-0.29	0.34
Depression	7.86 ± 3.88	8.95 ± 3.81	-2.031	243	0.043*	-2.13	-0.03	0.29
Interpersonal difficulty	8.03 ± 3.23	8.49 ± 3.32	-1.006	244	0.315	-1.36	0.44	0.14
Somatization	8.89 ± 4.61	10.32 ± 4.50	-2.262	239	0.025*	-2.68	-0.19	0.32
Total	37.79 ± 14.08	42.51 ± 13.60	-2.414	231	0.017*	-8.58	-0.87	0.35
PSS	21.53 ± 7.13	22.61 ± 5.22	-1.313	239	0.190	-2.70	0.54	0.18
RSES	26.97 ± 4.48	25.53 ± 4.96	2.154	242	0.032*	0.12	2.76	0.31
MSPSS	55.21 ± 17.23	53.51 ± 17.37	0.697	239	0.486	-3.09	6.48	0.10
Significant Other	17.91 ± 6.61	18.36 ± 6.35	-0.508	241	0.612	-2.22	1.31	0.07
Friends	18.21 ± 6.53	17.36 ± 6.72	0.922	244	0.357	-0.97	2.67	0.13
Family	18.95 ± 6.95	17.82 ± 6.90	1.166	242	0.245	-0.77	3.02	0.16
Truthful	3.31 ± 1.36	3.18 ± 1.28	4.101	240	< 0.001**	0.95	2.71	0.57
Perseverance	2.60 ± 1.05	2.32 ± 1.00	1.893	243	0.060	-0.01	0.68	0.27
Wisdom	15.72 ± 3.19	13.89 ± 3.23	3.716	242	< 0.001**	0.29	0.93	0.57
Generosity	3.69 ± 1.17	3.36 ± 1.32	2.156	242	0.032*	0.02	0.43	0.26
Five Precepts	3.25 ± 1.23	2.64 ± 1.16	1.795	243	0.074	-0.03	0.59	0.25
Meditation	1.67 ± 0.84	1.44 ± 0.70	1.564	243	0.119	-0.06	0.48	0.30
Tolerance	3.37 ± 1.14	3.09 ± 1.12	2.435	242	0.016*	0.07	0.66	0.35
Equanimity	3.09 ± 0.98	2.88 ± 0.97	1.929	243	0.055	-0.01	0.64	0.22
Determination	3.27 ± 1.00	2.90 ± 1.12	2.435	237	0.017*	0.082	0.653	0.35
Loving-Kindness	3.51 ± 1.07	3.19 ± 1.24	2.042	243	0.043*	0.10	0.626	0.27

OI-21 = Outcome Inventory-21, PSS = Perceived Stress Scale, RSES = Rosenberg Self-Esteem Scale, MSPSS = Multidimensional Scale of Perceived Social Support, iSBI = Inner Strength-Based Inventory, df = degree of freedom, *p < 0.05, **p < 0.001

There were no statistically significant differences in either negative or positive mental health outcomes between cat owners and non-pet owners.

## Discussion

This study investigated the relationship between pet ownership and mental health outcomes in university students with symptoms of borderline personality disorder. The main finding was that overall pet ownership was not significantly associated with reductions in negative mental health outcomes; however, we observed small but significant positive associations for particular psychological strengths, such as adherence to the Five Precepts and Wisdom (Cohen’s d = 0.29 and 0.27, respectively; both *p* < 0.002 after Bonferroni correction). Although effect sizes were small, these findings suggest that pet ownership may influence the development of inner strengths, even when the impact on symptoms such as depression or anxiety is limited.

When examining the impact of different types of pets, our data suggests that the kind of animal may play a role in the owner’s psychological well-being. Although cats were more commonly kept in this sample, we focused particular attention on dog ownership given its distinct patterns of interaction and emotional bonds. Compared with non-pet owners, dog ownership was associated with more favorable mental health outcomes. For example, dog ownership was linked to lower depression scores (Cohen’s d = 0.29), indicating a small but potentially meaningful effect. Dog owners also demonstrated higher levels of positive psychology, particularly truthfulness (Cohen’s d = 0.57) and wisdom (Cohen’s d = 0.53). These effect sizes, although modest, align with previous studies showing that regular engagement with dogs, including caring for, feeding, and walking, can foster a sense of life structure and a greater sense of purpose, both of which support mood regulation in young adults [17, 27, 28]. The effect size for depression, while small to moderate (d = 0.34), may still be meaningful clinically, especially given that even minor reductions in depression can improve functioning for students with high symptom loads. These findings reinforce previous observations that the responsibilities and routine involved in dog care support positive mental health.

Individuals with BPD often struggle with insecure attachment rooted in early relational traumas or inconsistent caregiving [17, 47]. These challenges manifest as difficulty trusting others, heightened fear of abandonment, and emotional dysregulation. As highlighted in this study, owning a dog may be uniquely therapeutic in mitigating these attachment-related struggles. Attachment theory posits that pets, particularly dogs, can act as a “secure base” due to their non-judgmental and consistently affectionate nature [14, 17, 20]. Dogs’ heightened responsiveness to human emotional needs provides a safe and predictable relationship for individuals with BPD [42, 45, 49], which can be invaluable for building trust and reducing fear of abandonment. Unlike human relationships, which may feel fraught with unpredictability, the unconditional acceptance offered by dogs can alleviate relational anxieties and foster emotional security [35]. Moreover, physical interactions with dogs, such as petting or grooming, have been shown to activate the parasympathetic nervous system, promoting relaxation and emotional calmness [29, 38, 49–50]. For individuals with BPD, who often experience intense emotional swings, these calming effects can help regulate overwhelming feelings of anger, sadness, or anxiety. However, it should be noted that the studies cited here predominantly focus on depression and anxiety in the general population ([14, 17, 30, 37]) rather than specifically on BPD. While there is overlap in emotional dysregulation between these conditions, the direct applicability of such findings to individuals with BPD requires cautious interpretation. Further research is needed to determine if the physiological or psychological effects seen in depression or generalized anxiety extend or manifest similarly in BPD populations. For this reason, the proposed pathways, such as stress reduction and emotional regulation through pet interaction, should be understood as potentially beneficial but not definitively established in BPD without additional targeted study. Moreover, in this manuscript, we acknowledge the distinction between the effects of pet ownership (the state of owning a pet) and pet attachment (the emotional bond or closeness felt toward one’s pet). As noted in recent literature, pet ownership does not always correspond to high attachment, and the psychological benefits may vary with the strength of pet attachment [32]. In this study, we primarily analyzed group differences by pet ownership status. Therefore, interpretations of the unique effects of strong pet–owner attachment, such as secure base formation or deep emotional support, should be made cautiously and warrant greater emphasis as an area for future research. To enhance clarity, we have specified in this paper whether results pertain to pet ownership or to pet attachment, as appropriate.

The bond between dog and owner can serve as a model for positive relationships, helping individuals with BPD develop interpersonal skills [17, 47, 51]. Dogs require attention, care, and consistent interaction, thereby encouraging owners to engage in nurturing behaviors that may generalize to human relationships. The structure provided by dog care fosters predictability and routine, which are critical for individuals with insecure attachments. This routine encourages responsibility and counters feelings of chaos often associated with BPD [17, 47, 49]. In addition, dogs act as social catalysts, encouraging owners to interact with others in dog-friendly environments and enhancing social confidence and perceived social support [18]. Finally, interacting with dogs encourages mindfulness, a skill that can reduce impulsive reactions and foster emotional stability. Dog ownership may elicit feelings of empathy and nurturance, which extend to self-compassion, reducing self-criticism and fostering self-acceptance [17, 46, 48].

Conversely, our findings showed no statistically significant or meaningful difference in either negative or positive mental health outcomes between cat owners and non-cat owners (for example, for depression, mean difference = 0.7, Cohen’s d = 0.09, *p* = 0.41), suggesting that cats’ less interactive nature may not offer the same psychosocial advantages as dog ownership, or that the effect is too subtle to detect with our measures [31–33]. As in the prior study, cat ownership was not significantly associated with depressive symptoms, and there was no significant association between cat attachment and mental health outcomes such as depression or anxiety [14, 34].

Regarding positive mental health outcomes, self-esteem, and some inner strengths were evident among dog-owner groups, suggesting that dog ownership may offer certain psychosocial benefits, such as companionship and a sense of responsibility, which have been linked to enhanced self-esteem [39], possibly due to positive social perceptions, a sense of respect, and validation from their pets [17, 36, 40]. In contrast, self-esteem levels among cat owners did not differ significantly from those of non-cat owners in our study, which may be due to cats’ generally less interactive nature [36].

It is important to contextualize these findings: although statistically significant effects were observed for several positive psychological outcomes among dog owners, effect sizes generally fell within the small-to-moderate range. This suggests that, although pet ownership, particularly dog ownership, may support certain aspects of well-being, its impact on mental health is modest rather than transformative. Nevertheless, for individuals struggling with emotional instability, even modest improvements in routine, social connection, and inner strengths may support recovery and improved daily functioning.

The design of this study limits the ability to establish a causal relationship. However, other related studies have shown that adults who own dogs score higher on self-empathy and are more conscientious than non-dog owners. Some data indicate that petting dogs also promotes mindfulness [17, 43–46]. Regarding the five precepts, this is readily understandable, as they promote kindness toward living beings, including animals. Closely related to loving kindness. Owning a dog may encourage a greater sense of kindness in the owner.

We compared each pet-ownership group only with “no-pet owners,” rather than with a reference group that included other pet owners. This approach reduces confounding and strengthens the clarity of our findings. Notably, most results remained highly consistent in both direction and magnitude, supporting the robustness of our conclusions. However, the change also highlighted additional positive results, notably a stronger, statistically significant association between pet ownership and increased tolerance. This suggests that using a homogenous “no-pet” reference group may better reveal unique benefits of pet ownership- effects which might be masked when comparisons are made against a heterogeneous reference group. These findings underscore the value of careful analytical choices in pet ownership research and reinforce the central observation that even small benefits associated with pet ownership, particularly in strengths such as tolerance, may have meaningful implications for student well-being.

Notably, the majority of participants in this study were single (98.0%). This demographic factor may have contributed to the provision of emotional support and companionship. For single individuals, pets may act as substitutes for close human relationships, potentially amplifying their impact on mental health and well-being. As such, the psychological benefits observed in our study may primarily reflect the experiences of single individuals, rather than those in committed romantic relationships. Future research should investigate the moderating effect of relationship status on the relationship between pet ownership and mental health outcomes.

In contrast to related studies, our results found no significant difference in perceived social support between pet owners and non-owners, including dog owners. This could contribute to differences in the study design. A study revealed that individuals who had pets during childhood reported higher levels of social support [41]. One possible explanation for the differing results in our study is the use of a different measure for assessing social support. Additionally, our study did not account for variations in attachment levels, academic years, or sex, all of which could influence perceived social support. Especially among dog owners, prior research shows that dog owners experience greater attachment and perceived social support than owners of other pets, such as birds or reptiles [42], because dogs are more likely than other pets to form social connections within their communities [31–32]. A previous study has shown a significant association between pet attachment and perceived social support [42].

The present study provides nuanced guidance for mental health professionals working with university students showing symptoms of borderline personality disorder (BPD). Our updated results indicate that while pet ownership overall was not associated with lower adverse mental health outcomes, dog owners specifically reported significantly lower levels of anxiety, depression, and somatization, as well as higher self-esteem, when compared to those without pets. Additionally, pet owners—particularly dog owners—demonstrated significantly higher levels of several key inner strengths, including truthfulness, wisdom, generosity, tolerance, determination, and loving-kindness. These findings suggest that the presence of a dog may play a supportive role in fostering not only emotional well-being but also the development of positive personal attributes. While pet ownership should not be considered a substitute for evidence-based clinical care, clinicians may find it valuable to inquire about and incorporate companion-animal relationships into assessment and intervention planning, especially for clients who may benefit from enhanced routines, emotional support, and the cultivation of inner strengths.

Future research should employ qualitative methodologies to gain deeper insight into how students with BPD symptoms experience and attribute meaning to their relationships with pets. Qualitative approaches could illuminate the specific mechanisms by which pet companionship, particularly with dogs, supports psychological well-being and the development of inner strengths among young adults, thereby informing therapeutic models and interventions.

### Strengths and limitations

This study provides significant contributions to understanding the role of pet ownership in mental health, particularly among university students with symptoms of BPD. These findings suggest that pet ownership, particularly with dogs, may enhance specific positive mental health outcomes. This research emphasizes the unique psychological benefits of pets and suggests non-traditional strategies to support mental health. Additionally, the findings align with existing theories about the therapeutic effects of pets, suggesting that dogs, in particular, foster emotion regulation and provide a sense of purpose, which is critical for individuals with emotional instability. These insights are valuable for expanding mental health care approaches, particularly through interventions such as pet-assisted therapy.

Like other studies in this area, this investigation has limitations that must be acknowledged. First, a cross-sectional design precludes establishing causality between pet ownership and mental health outcomes, as it only captures associations at a single point in time. Longitudinal research is needed to explore the potential long-term effects of pet ownership on mental health. Second, this study did not assess attachment levels or interaction frequency with pets, both of which could play significant roles in mental health outcomes. Third, confounding factors, such as living arrangements, physical activity, and other social supports, were uncontrolled and may have influenced the results. Fourth, the inclusion of participants with other personality disorders complicates interpretation, as these co-occurring conditions could affect mental health outcomes differently. Finally, reliance on self-reported data introduces potential biases in how participants perceive and report on their mental health. Addressing these limitations in future research would help clarify the role of pet ownership in supporting mental health, especially in individuals with BPD symptoms.

## Conclusion

In summary, this study examined the effects of pet ownership on mental health outcomes in university students with symptoms of borderline personality disorder (BPD). The results indicate that, although overall pet ownership does not significantly affect adverse mental health outcomes, dog ownership may confer unique psychological benefits. Dog owners reported significantly lower levels of anxiety, depression, and somatic symptoms compared to non-pet owners. Moreover, dog owners demonstrated higher self-esteem and inner strengths, as reflected in scores for truthfulness, wisdom, generosity, tolerance, determination, and loving-kindness.

These findings align with existing literature suggesting that consistent physical and emotional interactions with dogs may foster emotional attachments, improve emotion regulation, and strengthen coping skills—factors that are particularly valuable for individuals with BPD symptoms. Conversely, cat ownership was not significantly associated with mental health benefits, potentially reflecting the more independent and less interactive nature of cats.

Overall, the type of pet and the quality of the owner-pet relationship may play pivotal roles in influencing mental health outcomes. Further research should explore these relationships, considering attachment levels and individual personality variables, to clarify the nuances of how pet ownership impacts mental health in university students with BPD symptoms.

## Data Availability

The data supporting the findings of this study are available from the corresponding author upon reasonable request due to privacy and ethical restrictions.

## Abbreviations

BPD: Borderline personality disorder
DSM-5: Diagnostic and Statistical Manual of Mental Disorders, Fifth Edition
OI-21: Outcome Inventory-21
RSES-TR: Rosenberg Self-Esteem Scale-Thai Revised
R-Thai MSPSS: Revised Thai version of the Multidimensional Scale of Perceived Social Support
T-PSS-10: Thai version of the 10-item Perceived Stress Scale
iSBI: Inner Strength-Based Inventory
SI-Bord: Screening Instrument for Borderline Personality Disorder

## References

[1] American Psychiatric Association. Diagnostic and statistical manual of mental disorders: DSM-5-TR. 5th ed. Washington, DC: American Psychiatric Association Publishing; 2022.

[2] Wang Y, Zhou X, Wang Z, Yang H, Zhang Y. Borderline personality symptoms in Chinese university students: prevalence and associations with emotion dysregulation and interpersonal problems. Psychiatry Res. 2023;326:115238.

[3] Lohanan T, et al. Development and validation of a screening instrument for borderline personality disorder (SI-Bord) for use among university students. BMC Psychiatry. 2020;20(1):479. 32998759 10.1186/s12888-020-02807-6 PMC7526163

[4] Tomko RL, et al. Characteristics of borderline personality disorder in a community sample: comorbidity, treatment utilization, and general functioning. J Pers Disord. 2014;28(5):734–50. 25248122 10.1521/pedi_2012_26_093 PMC3864176

[5] Arnett JJ, Žukauskienė R, Sugimura K. The new life stage of emerging adulthood at ages 18–29 years: implications for mental health. Lancet Psychiatry. 2014;1(7):569–76. 26361316 10.1016/S2215-0366(14)00080-7

[6] Soloff PH, et al. Characteristics of suicide attempts of patients with major depressive episode and borderline personality disorder: a comparative study. Am J Psychiatry. 2000;157(4):601–8. 10739420 10.1176/appi.ajp.157.4.601

[7] Wongpakaran N et al. Borderline personality symptoms: what not to be overlooked when approaching suicidal ideation among university students. Healthcare (Basel). 2021;9(10). 10.3390/healthcare9101399 PMC8535964 34683078

[8] Grilo CM, Udo T. Association of borderline personality disorder criteria with suicide attempts among US adults. JAMA Netw Open. 2021;4(5):e219389. 33974054 10.1001/jamanetworkopen.2021.9389 PMC8114135

[9] Levy KN. The implications of attachment theory and research for Understanding borderline personality disorder. Dev Psychopathol. 2005;17(4):959–86. 16613426 10.1017/s0954579405050455

[10] Kaurin A, et al. Attachment and borderline personality disorder: differential effects on situational Socio-Affective processes. Affect Sci. 2020;1(3):117–27. 33718882 10.1007/s42761-020-00017-7 PMC7954219

[11] Liebke L, et al. Loneliness, social networks, and social functioning in borderline personality disorder. Personal Disord. 2017;8(4):349–56. 27505189 10.1037/per0000208

[12] Bornstein RF, et al. Interpersonal dependency in borderline personality disorder: clinical context and empirical evidence. J Pers Disord. 2010;24(1):109–27. 20205501 10.1521/pedi.2010.24.1.109

[13] Perry JC, Klerman GL. Clinical features of the borderline personality disorder. Am J Psychiatry. 1980;137(2):165–73. 7352571 10.1176/ajp.137.2.165

[14] Zebrowska M, et al. Pet attachment and anxiety and depression in Middle-Aged and older women. JAMA Netw Open. 2024;7(8):e2424810. 39088217 10.1001/jamanetworkopen.2024.24810 PMC11294964

[15] Carr S, Rockett B. Fostering secure attachment: experiences of animal companions in the foster home. Attach Hum Dev. 2017;19(3):259–77. 28277096 10.1080/14616734.2017.1280517

[16] Hui Gan GZ, et al. Pet ownership and its influence on mental health in older adults. Aging Ment Health. 2020;24(10):1605–12. 31242754 10.1080/13607863.2019.1633620

[17] Hawkins RD, Kuo CH, Robinson C. Young adults’ views on the mechanisms underpinning the impact of pets on symptoms of anxiety and depression. Front Psychiatry. 2024;15:1355317. 38425998 10.3389/fpsyt.2024.1355317 PMC10902138

[18] Wood L, et al. The pet factor–companion animals as a conduit for getting to know people, friendship formation and social support. PLoS ONE. 2015;10(4):e0122085. 25924013 10.1371/journal.pone.0122085 PMC4414420

[19] Richie FJ, et al. Social support and suicidal ideation among prisoners with major depressive disorder. Arch Suicide Res. 2021;25(1):107–14. 31369343 10.1080/13811118.2019.1649773 PMC7067664

[20] Ein N, Li L, Vickers K. The effect of pet therapy on the physiological and subjective stress response: A meta-analysis. Stress Health. 2018;34(4):477–89. 29882342 10.1002/smi.2812

[21] Hayden-Evans M, Milbourn B, Netto J. Pets provide meaning and purpose’: a qualitative study of pet ownership from the perspectives of people diagnosed with borderline personality disorder. Adv Mental Health. 2018;16(2):152–62.

[22] Wongpakaran N, Wongpakaran T, Kövi Z. Development and validation of 21-item outcome inventory (OI-21). Heliyon. 2022;8(6):e09682. 35711988 10.1016/j.heliyon.2022.e09682 PMC9193908

[23] Wongpakaran T, Wongpakaran N. A comparison of reliability and construct validity between the original and revised versions of the Rosenberg Self-Esteem scale. Psychiatry Investig. 2012;9(1):54–8. 22396685 10.4306/pi.2012.9.1.54 PMC3285741

[24] Wongpakaran T, Wongpakaran N, Ruktrakul R. Reliability and validity of the multidimensional scale of perceived social support (MSPSS): Thai version. Clin Pract Epidemiol Ment Health. 2011;7:161–6. 22114620 10.2174/1745017901107010161 PMC3219878

[25] Wongpakaran N, Wongpakaran T. The Thai version of the PSS-10: an investigation of its psychometric properties. Biopsychosoc Med. 2010;4:6. 20540784 10.1186/1751-0759-4-6 PMC2905320

[26] Wongpakaran N, Wongpakaran T, Kuntawong P. Development and validation of the (inner) Strength-Based inventory. Mental Health Relig Cult. 2020;23(3–4):263–73.

[27] Applebaum JW, et al. The impact of pets on everyday life for older adults during the COVID-19 pandemic. Front Public Health. 2021;9:652610. 33898382 10.3389/fpubh.2021.652610 PMC8062698

[28] Musich S, et al. Purpose in life and positive health outcomes among older adults. Popul Health Manag. 2018;21(2):139–47. 28677991 10.1089/pop.2017.0063 PMC5906725

[29] Moretti F, et al. Pet therapy in elderly patients with mental illness. Psychogeriatrics. 2011;11(2):125–9. 21707862 10.1111/j.1479-8301.2010.00329.x

[30] Ambrosi C, et al. Randomized controlled study on the effectiveness of animal-assisted therapy on depression, anxiety, and illness perception in institutionalized elderly. Psychogeriatrics. 2019;19(1):55–64. 30221438 10.1111/psyg.12367

[31] Menchetti L et al. My dog is not my cat: owner perception of the personalities of dogs and cats living in the same household. Animals (Basel). 2018;8(6). 10.3390/ani8060080 PMC6025356 29882930

[32] Serpell JA. Evidence for an association between pet behavior and owner attachment levels. Appl Animals Behav Sci. 1996;47(1):49–60.

[33] González-Ramírez MT, Landero-Hernández R. Pet-Human relationships: dogs versus cats. Animals (Basel). 2021;11(9). 10.3390/ani11092745 PMC8470704 34573712

[34] Sharpley C, et al. Pet ownership and symptoms of depression: A prospective study of older adults. J Affect Disord. 2020;264:35–9. 31846900 10.1016/j.jad.2019.11.134

[35] Fiori G, et al. The challenge of pet therapy in systemic sclerosis: evidence for an impact on pain, anxiety, neuroticism and social interaction. Clin Exp Rheumatol. 2018;36(4):135–41. 30277859

[36] Schulz C, König HH, Hajek A. Differences in Self-Esteem between Cat Owners, dog Owners, and individuals without pets. Front Vet Sci. 2020;7:552. 32984412 10.3389/fvets.2020.00552 PMC7492270

[37] Rahmani N, Barazandeh A, Sepehrtaj S. Psychological profile of pet owners in Isfahan, Iran. Brazilian J Veterinary Res Animals Sci. 2021;58:e179974.

[38] Kertes DA, et al. Effect of pet dogs on children’s perceived stress and cortisol stress response. Soc Dev. 2017;26(2):382–401. 28439150 10.1111/sode.12203 PMC5400290

[39] Purewal R et al. Companion animals and Child/Adolescent development: a systematic review of the evidence. Int J Environ Res Public Health, 2017;14(3). 10.3390/ijerph14030234 PMC5369070 28264460

[40] Liu M, Wu L, Ming Q. How does physical activity intervention improve Self-Esteem and Self-Concept in children and adolescents? Evidence from a Meta-Analysis. PLoS ONE. 2015;10(8):e0134804. 26241879 10.1371/journal.pone.0134804 PMC4524727

[41] Barker SB, et al. The relationship between pet Ownership, social Support, and internalizing symptoms in students from the first to fourth year of college. Appl Dev Sci. 2020;24(3):279–93. 32742161 10.1080/10888691.2018.1476148 PMC7394412

[42] Bekker OA, Mallavarapu S. Pet attachment and the social support that pets provide to college students. J Undergrad Res. 2019;6:4.

[43] McConnell AR, et al. Friends with benefits: on the positive consequences of pet ownership. J Pers Soc Psychol. 2011;101(6):1239–52. 21728449 10.1037/a0024506

[44] Oliva JL, Johnston KL. Puppy love in the time of corona: dog ownership protects against loneliness for those living alone during the COVID-19 lockdown. Int J Soc Psychiatry. 2021;67(3):232–42. 32701015 10.1177/0020764020944195 PMC7383093

[45] Cloutier A, Peetz J. Relationships’ best friend: links between pet Ownership, Empathy, and romantic relationship outcomes. Anthrozoös. 2016;29(3):395–408.

[46] Daly B, L Morton L. Empathic differences in adults as a function of childhood and adult pet ownership and pet type. Anthrozoos: Multidisciplinary J Interact People Anim. 2009;22:371–82.

[47] Balluerka N, et al. Influence of animal-assisted therapy (AAT) on the attachment representations of youth in residential care. Child Youth Serv Rev. 2014;42:103–9.

[48] Jiang H, et al. The influence of pet ownership on self-compassion among nurses: a cross-sectional study. PeerJ. 2023;11:e15288. 37159831 10.7717/peerj.15288 PMC10163869

[49] Riddoch KA, Hawkins RD, Cross ES. Exploring behaviours perceived as important for human—Dog bonding and their translation to a robotic platform. PLoS ONE. 2022;17(9):e0274353. 36170337 10.1371/journal.pone.0274353 PMC9518860

[50] Beetz A, et al. Psychosocial and Psychophysiological effects of human-animal interactions: the possible role of Oxytocin. Front Psychol. 2012;3:234. 22866043 10.3389/fpsyg.2012.00234 PMC3408111

[51] Faner JMV, et al. Pet attachment and prosocial attitude toward humans: the mediating role of empathy to animals. Front Psychol. 2024;15:1391606. 38933589 10.3389/fpsyg.2024.1391606 PMC11200204

[52] Calder R, Dakin P. Valued, loved and safe: the foundations for healthy individuals and a healthier society. Med J Aust. 2023;219:S11–4. 37982336 10.5694/mja2.52135

[53] O’Hagan AD, Gavigan N, Goss H. The PAWS project – Promoting and supporting the wellbeing of undergraduate students with service dogs. Human-Animal Interact. 2024;12(1):1–7.

[54] Kirnan JP, Shapiro AR, Mistretta AJ, Sellet M, Fotinos G, Blair B. Emotional support animals supporting college students’ mental health and well-being: a qualitative analysis exploring practices, policies, and perceptions. J Am Coll Health. 2022;1–11. 10.1080/07448481.2022.2095871 35816762

